# Dynamics of dichoptic masking in the primary visual cortex

**DOI:** 10.1186/1471-2202-15-S1-P145

**Published:** 2014-07-21

**Authors:** Eva Chadnova, Alexandre Reynaud, Simon Clavagnier, Daniel H Baker, Sylvain Baillet, Robert F Hess

**Affiliations:** 1 McGill Vision Research Unit, McGill University, Montreal, Quebec, H3A2T5, Canada; 2 McConnell Brain Imaging Center, Montreal Neurological Institute, McGill University, Montreal, Quebec, H3A2B4, Canada; 3 Department of Psychology, University of York, Heslington, York, YO10 5DD, UK

## 

The inputs from the two eyes interact in a nonlinear fashion. This interaction can be either excitatory or inhibitory: Excitatory interaction (combination) occurs first in the primary visual cortex but little is known about the site of the inhibitory interaction (suppression). To investigate the latter, experimental paradigms typically present distinct inputs to the eyes (dichoptic presentation, one target and one mask are respectively presented to different eyes at the same time). Here we used magnetoencephalography (MEG) source imaging to establish the site of the cortical neural signature of interocular suppression in visual cortex.

We presented different noise stimuli to each eye: The target-noise was presented for contrasts ranging between 0 and 32 %. The mask-contrast was presented to the other eye at fixed contrast (32%). We flickered the two noise stimuli (4 and 6 Hz) to elicit a frequency-tagged steady-state visual evoked response (SSVER) that was readily detectable in MEG traces [1]. Four participants passively observed the visual presentation while keeping their gaze fixed on the center of the screen. The Brainstorm application was used to analyze the MEG data [3]. MEG source time series were extracted from cortical regions of interest (ROIs) defined from the visual retinotopic maps of each participant obtained from previous fMRI acquisitions.

Using the power of the cortical responses to the frequency-tagged stimuli, we constructed contrast response functions for all the ROIs (Figure 1 A). To investigate dynamics of propagation of the response along visual cortical areas, the instantaneous phase of the signal was identified in each ROI. As expected [2], when the target was presented alone, the power of the responses was found to increase monotonically with contrast (Figure 1 B, solid line). When a mask was added to the other eye, the contrast response was attenuated (Figure 1 B, dotted line). Interestingly, the mask presented at a fixed contrast was also found to be gradually suppressed with increasing target contrast (Figure 1 C). These effects were revealed for responses as early as the primary visual cortex. In the time-domain, we detected a progressive phase shift between the cortical responses along the ventral and dorsal streams.

**Figure 1:**
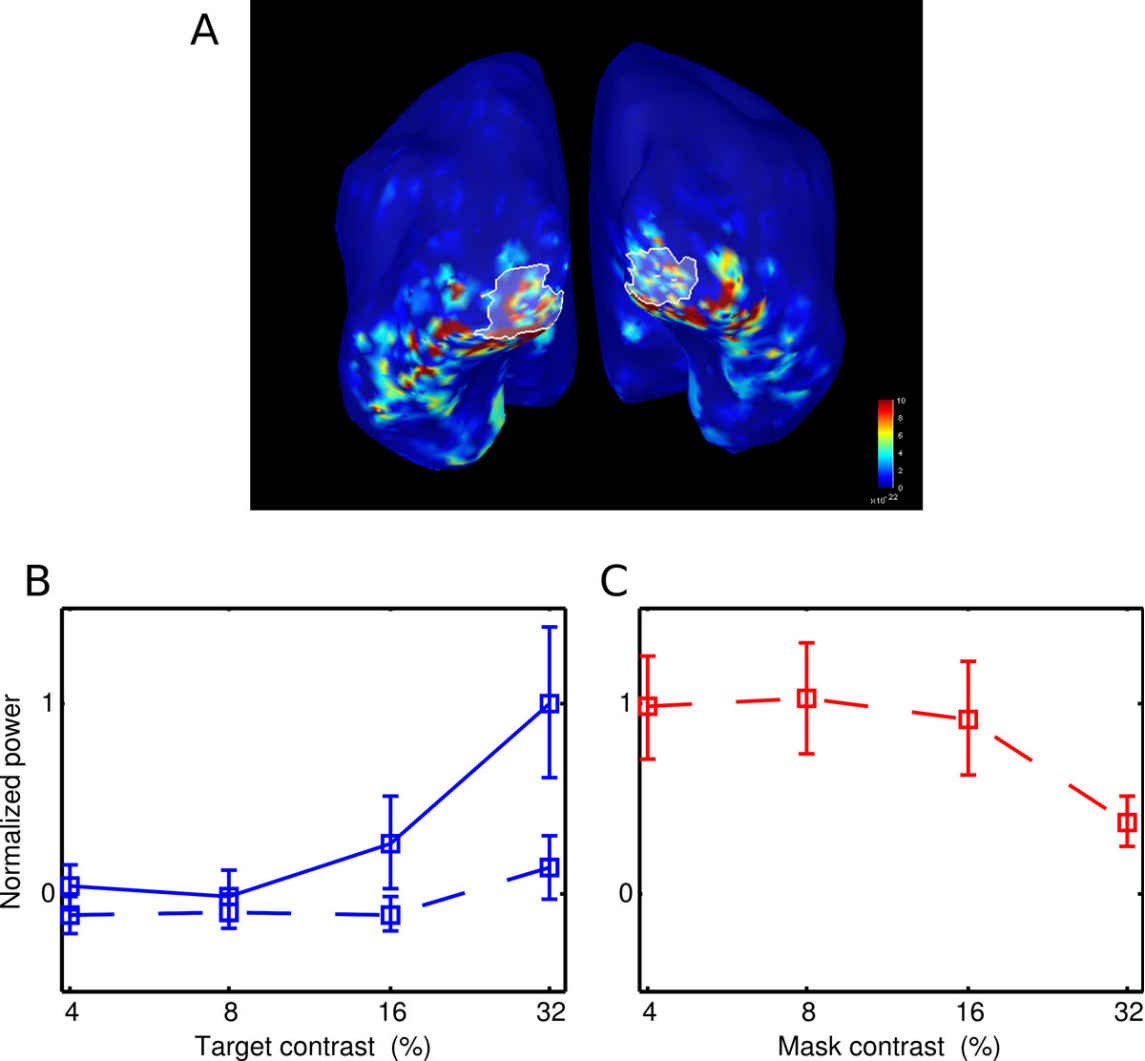
**A**. Localization of V1 on the activity map **B.** Normalized power of SSVER response as a function of contrast for target only (solid line), target and mask (dotted line); **C.** Normalized power in a 32% contrast mask as the contrast in the target is increasing.

## Conclusions

We characterized dichoptic suppression in the visual cortex with MEG. This suppression occurs as early as the primary visual cortex. The suppression between the inputs of varying contrast was also well defined in the MEG power signal. The temporal resolution of MEG cortical imaging enables the analysis of the phase shifts and delay of the steady-state visual-evoked response between cortical regions. When combined with individual visual cortical mapping, our method provides a temporally and spatially precise tool for the detailed elucidation of suppression in the visual processing induced by dichoptic masking.
